# Lactose intolerance

**DOI:** 10.1093/emph/eoaa006

**Published:** 2020-02-17

**Authors:** Andrea S Wiley

**Affiliations:** Department of Anthropology, Indiana University, 130 Student Building, Bloomington, IN 47405, USA

**Keywords:** Lactose intolerance, lactase, milk, biological normalcy

## LACTOSE INTOLERANCE

Lactose intolerance results from incomplete digestion of the milk sugar lactose. Primary lactose intolerance is characterized by gastrointestinal discomfort from osmotic diarrhoea and colonic bacterial fermentation of lactose. Lactose is found only in mammalian milk and requires the intestinal enzyme lactase to cleave it into glucose and galactose, which can be absorbed.

Wild, non-human mammals would not have encountered lactose in their diet after weaning, and lactase production ceases around weaning age [1]. This is lactase non-persistence (LNP). Most humans follow this pattern, and hence may experience lactose intolerance if they consume milk or high-lactose dairy products, although there is individual variation in symptomatology. In contrast, populations of European or mainly pastoralist ancestry generally have higher frequencies of lactase persistence (LP; continued production of lactase), and are less likely to experience lactose intolerance [2].

The genetic basis for population variation in lactase production is well-described although not yet complete, with mutations responsible for LP identified in a regulatory area upstream from the lactase gene [2].

## EVOLUTIONARY PERSPECTIVES

**Figure 1.**
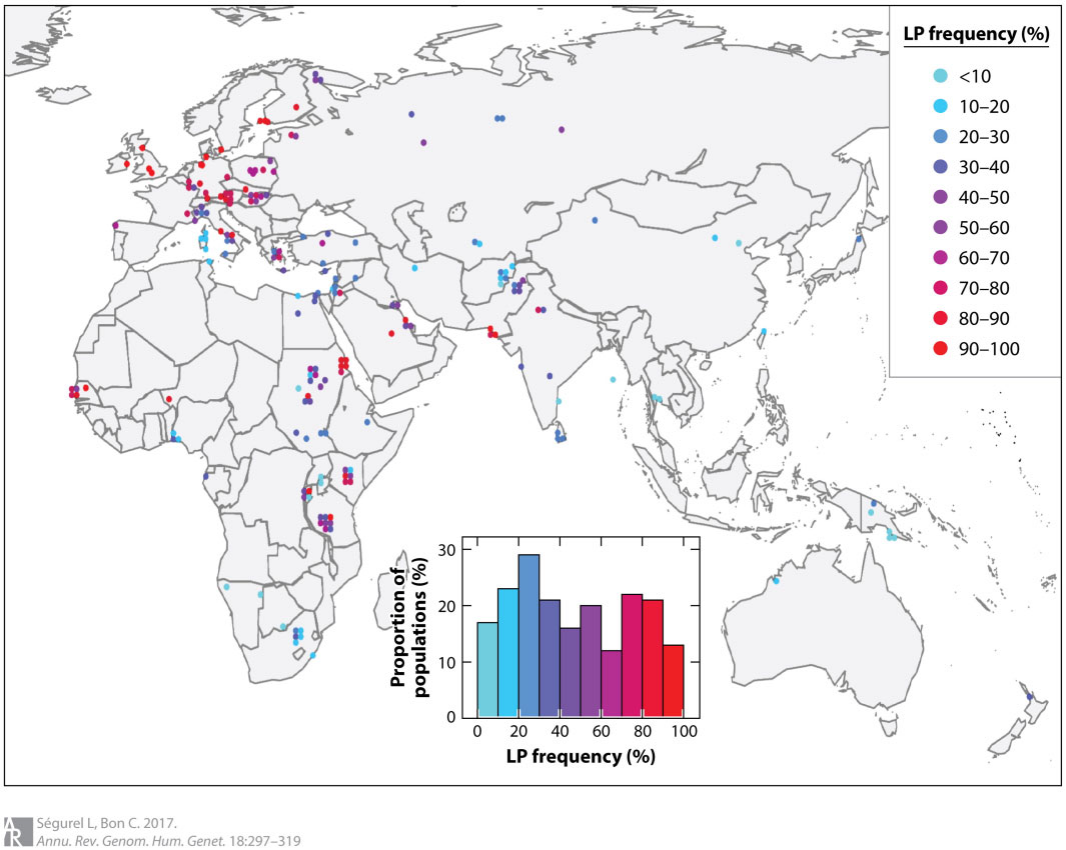
Geographic distribution and frequency of LP alleles.

LNP is the ancestral state for humans. LP is found almost exclusively in populations with long dairying histories (Fig. 1), and LP alleles spread after dairy animal domestication in the Old World (∼10 000 ya) [3].

Not all dairying populations show high LP frequencies, as they consume dairy products with reduced lactose content (e.g. cheese, yogurt) [2].

LP alleles show strong signals of selection [4] and also have spread through migration [5]. Hypotheses advanced to understand why LP spread among some dairying populations include the following [reviewed in 6, 7]:

- Lifelong access to nutrient-rich milk;
- Lifelong access to a carbohydrate and fluid source, critical to pastoralists living in hot, arid environments;
- Lactose can enhance calcium absorption, which may be compromised by low Vitamin D synthesis in high latitude environments;
- Human consumption of bovine milk may accelerate reproductive maturation or physical growth, or contribute to larger adult size, possibly due to milk’s stimulatory effects on insulin-like growth factor-I [7].

Given the diversity of environments in which LP populations live, a universal explanation is unlikely. For non-dairying groups, the above hypotheses suggest that LP mutations did not spread as they did not confer an advantage.

## FUTURE IMPLICATIONS

Most humans are LNP and at risk for lactose intolerance when following dietary guidelines, which commonly recommend milk. Milk consumption beyond weaning was not practiced for most of our species’ evolution, or in most historical populations.

Clinicians should avoid frequently-used terms such as ‘lactase deficiency’ or ‘lactose maldigestion’ to describe the cause of lactose intolerance, and instead use LNP, which does not imply pathology, and recognize that LNP is the common form, rather than the exception, among humans [8].
